# ^99m^Tc-MIBI SPECT/CT imaging had high sensitivity in accurate localization of parathyroids before parathyroidectomy for patients with secondary hyperparathyroidism

**DOI:** 10.1080/0886022X.2019.1662804

**Published:** 2019-09-20

**Authors:** Ming Zeng, Wei Liu, Xiaoming Zha, Shaowen Tang, Jin Liu, Guang Yang, Huijuan Mao, Xiangbao Yu, Bin Sun, Bo Zhang, Chun Ouyang, Lina Zhang, Jing Guo, Jing Wang, Yaoyu Huang, Yogendranath Purrunsing, Hanyang Qian, Ningning Wang, Changying Xing

**Affiliations:** a Department of Nephrology, The First Affiliated Hospital of Nanjing Medical University, Jiangsu Province Hospital, Nanjing, China;; b Department of Nuclear Medicine, The First Affiliated Hospital of Nanjing Medical University, Jiangsu Province Hospital, Nanjing, China;; c Department of General Surgery, The First Affiliated Hospital of Nanjing Medical University, Jiangsu Province Hospital, Nanjing, China;; d Department of Epidemiology and Biostatistics, School of Public Health, Nanjing Medical University, Nanjing, China;; e Clinical Medicine Research Institution, The First Affiliated Hospital of Nanjing Medical University, Jiangsu Province Hospital, Nanjing, China;; f Department of Nephrology, Henan Provincial Key Laboratory of Kidney Disease and Immunology, Henan Provincial People’s Hospital, People's Hospital of Zhengzhou University, Zhengzhou, China

**Keywords:** Secondary hyperparathyroidism, parathyroid, parathyroidectomy, dual-phase ^99m^Tc-MIBI scintigraphy, single-photon emission computed tomography/computed tomography imaging

## Abstract

**Purpose:** Accurate preoperative parathyroid localization is important for successful parathyroidectomy (PTX). The aim of our study was to investigate whether SPECT/CT has enhanced effect in preoperative localization of parathyroids.

**Methods:** In our retrospective cohort study, we evaluated the effects of technetium-99m methoxyisobutylisonitrile-single-photon emission computed tomography/computed tomography (^99m^Tc-MIBI SPECT/CT) on preoperative parathyroid localization for 645 secondary hyperparathyroidism (SHPT) patients. Among them, 569 successful PTX patients were divided into group A (received ^99m^Tc-MIBI scintigraphy, *n* = 175) and group B (received ^99m^Tc-MIBI scintigraphy and SPECT/CT imaging, *n* = 394). Sensitivity, specificity, and consistency of two imaging methods in preoperative localization of parathyroids were compared.

**Results:** Overall sensitivity and consistency were higher in group B, while there was no difference in specificity between the two groups. In group A, the sensitivity of ^99m^Tc-MIBI was 50.00%, 77.11%, 61.76%, and 76.54% in the right upper gland (RU), right lower gland (RL), left upper gland (LU), and left lower gland (LL) subgroups, while the consistency was 52.00%, 76.57%, 61.71%, and 75.43%, respectively. In group B, the sensitivity of ^99m^Tc-MIBI with SPECT/CT was 69.39%, 90.03%, 78.07%, and 84.27%, and the consistency was 69.54%, 88.32%, 78.43%, and 84.26%, respectively. The sensitivity and consistency in lower glands were higher than in upper glands in both groups. Sensitivity for eutopic parathyroid was higher in group B, while there was no difference for ectopic parathyroid.

**Conclusions:** ^99m^Tc-MIBI SPECT/CT can increase the sensitivity and consistency of preoperative localization of eutopic parathyroid glands, and it can accurately locate ectopic parathyroid without sensitivity improvement.

## Introduction

Secondary hyperparathyroidism (SHPT) is an intractable disease of patients with chronic kidney disease (CKD), leading to substantial morbidity or mortality [1,2]. Diet regulation, sufficient hemodialysis, and medical treatment are effective in most cases [3].

However, resistance to medical treatment and insisting symptoms may necessitate surgical treatment in 10% of patients [3]. Surgical resection of all hyperplastic or ectopic glands is necessary for successful parathyroidectomy (PTX), but it is difficult to resect all parathyroid glands because of the existence of supernumerary and ectopic parathyroids. The occurrence of persistent SHPT after PTX is reported to be 0.4–25% [4–6].

Developmental defects occurring at an early stage of embryogenesis can result in the location of parathyroid glands in ectopic sites. Ectopic parathyroids are a common cause of surgical failure and concomitant persistent hyperparathyroidism [4,7]. The prevalence of ectopic parathyroids ranges from 28% to 42.8% in autopsy series [8,9], although even lower rates (2%) have been reported [10], and 35.1–45.7% in SHPT with end-stage renal disease (ESRD) [11–13]. Therefore, accurate preoperative parathyroid localization is very important.

Dual-phase technetium-99m methoxyisobutylisonitrile (^99m^Tc-MIBI) is currently the most common diagnostic examination performed for preoperative parathyroid localization. The contrast agent of ^99m^Tc-MIBI, which is injected intravenously and absorbed in the thyroid and hyperparathyroid glands, can be detected by a gamma camera. Since the clearance rate of the thyroid is much faster than that of the parathyroid glands, as time elapses, the uptake of the imaging agent ratio in the hyperparathyroid glands increases, showing hyperplastic parathyroids.

Since 1999, ^99m^Tc-MIBI imaging has been applied in our hospital (the First Affiliated Hospital of Nanjing Medical University) for preoperative localization of parathyroid glands in SHPT patients [14]. The method has high sensitivity and specificity, but still has some limitations such as the presence of P-glycoprotein or other efflux proteins which might cause rapid tracer washout and reduced sensitivity [15]. Furthermore, the sensitivity of ^99m^Tc-MIBI for ectopic parathyroid glands is high, but the anatomical localization is not accurate enough. Since July 2013, single-photon emission computed tomography/computed tomography (SPECT/CT) has been applied along with dual-phase ^99m^Tc-MIBI planar imaging in our hospital. The aim of our study was to investigate whether SPECT/CT has enhanced effect in preoperative localization of parathyroids.

## Materials and methods

### Study population

The population of this historical cohort study consisted of 637 CKD patients with SHPT who underwent PTX in the First Affiliated Hospital of Nanjing Medical University (Jiangsu Province Hospital) from March 2010 to December 2016. Those who received successful operations were enrolled in further study. Preoperatively, patients in group A only received dual-phase ^99m^Tc-MIBI planar imaging from March 2010 to June 2013, and patients in group B received dual-phase ^99m^Tc-MIBI planar imaging plus early phase SPECT/CT from July 2013 to December 2016.

PTX was performed in severe SHPT patients who failed to respond to medical therapy [16]. Our surgical indications included: persistent serum intact parathyroid hormone (iPTH) >800 pg/mL; hypercalcemia and/or hyperphosphatemia that could not be controlled by medical therapy; obvious clinical manifestations such as bone pain, pruritus, ectopic calcification or fracture; and at least one enlarged parathyroid gland discovered by ^99m^Tc-MIBI scan or SPECT/CT [4,16].

**Figure 1.**
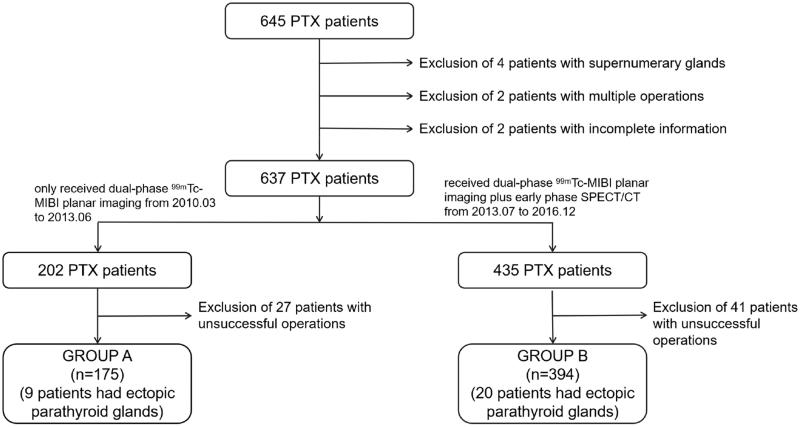
Flow chart of the study population.

Of the 645 patients, 4 (0.6%) patients excluded because of supernumerary glands, 2 (0.3%) patients excluded because of multiple operations and 2(0.3%) patients excluded because of incomplete information. Another 68 (10.5%) patients were also excluded from the analysis because of unsuccessful operations. As a result, 569 patients were considered for further analysis (Figure 1).

### Determinations of blood parameters

Routine blood tests were performed by an LH-750 Hematology Analyzer (Beckman Coulter, Fullerton, CA). Biochemical indices were measured with an automatic biochemical analyzer (AU5400; Olympus, Tokyo, Japan). Serum iPTH levels were measured by a UniCel DxI800 Access Immunoassay System (Beckman Coulter Inc., Fullerton, CA). The recommended range of serum iPTH was 12–88 pg/mL among healthy people.

All participants gave informed consent, which was approved by the Research Ethics Committee of the First Affiliated Hospital of Nanjing Medical University, People’s Republic of China (2019-SR-051). All investigations were conducted in accordance with the Declaration of Helsinki.

### ^99m^Tc-MIBI imaging protocol

Patients in group A received dual-phase ^99m^Tc-MIBI planar imaging, which consisted of planar images of the neck and upper thorax obtained 10 min (early imaging) and 2 h (delayed imaging) after intravenous injection of 740MBq ^99m^Tc-MIBI, with 128 × 128 matrix, zoom 2.57, and each frame acquisition counts 1000 k.

Patients in group B received dual-phase ^99m^Tc-MIBI planar imaging plus early phase SPECT/CT imaging. The SPECT dual detectors (Siemens, Germany, Symbia T6) were placed at the 180° position and rotated 180° around the patients, with the field of view covering the patient’s neck and upper chest. A total of 48 frames (20 s/frame, matrix 128 × 128, zoom 1.0) were acquired. After the completion of SPECT acquisition, the bed was moved forward to the CT scan position, matching the CT scan field of view with the SPECT imaging field of view, and then CT transmission scan was performed (CT acquisition and reconstruction conditions: 120 kV, 80 mA, matrix 512 × 512, layer thickness 3.75 mm, pitch 1). For image reconstruction, Ordered Subsets Expectation Maximization (OSEM) iteration technology was used. Image analysis was focused with high radioactivity distribution on the early or delayed phase planar images or SPECT/CT images, excluding other image interferences was judged as positive. Interpretation of ^99m^Tc-MIBI scintigraphy was performed in consensus by two experienced nuclear medicine physicians. The image findings were scored as positive or negative.

### Definition of ectopic parathyroid glands

Hyperplastic parathyroid glands located inside the superior mediastinum and thyroid were regarded as ectopic parathyroid glands. The incidence and sensitivity of ectopic parathyroid glands in the two subgroups were compared.

## Surgical procedures

Total parathyroidectomy (tPTX) with autotransplantation was performed routinely under general anesthesia in all SHPT patients. All operations were performed by the same surgeon.

Intraoperative frozen section analysis was routinely adopted to verify that the resected specimen was parathyroid tissue. The selected diffuse hyperplasia parathyroid fragment was cut into slices of about 1 mm^3^, and eight slices were transplanted into forearm muscles without an arteriovenous fistula for hemodialysis. After surgery, pathological sections were examined carefully. Hyperplastic parathyroid glands resected in operation were confirmed by postoperative pathology. The scintigraphy finding for each gland was defined as true positive, false positive, true negative, or false negative on the basis of the pathology results. Comparisons of sensitivity, specificity and consistency between the two groups were made according to the parathyroid pathology results.

### Definition of successful PTX

Here we adopted the criterion based on the work of Stracke and our group [4,17]. Postoperative iPTH levels were measured on the first and fourth postoperative days. Patients with serum iPTH <50 pg/mL at least once in the first postoperative week were defined as successful PTX. Patients with serum iPTH >50 pg/mL twice in the first postoperative week were followed up to verify the effect of surgery. Depending on serum iPTH values within 6 months, patients with iPTH <300 pg/mL were regarded as the successful PTX group, and those whose iPTH >300 pg/mL were classified as persistent SHPT.

### Statistical analysis

Data were analyzed using SPSS software (version 22.0; Chicago, IL). Continuous variables were presented as mean ± SD or median (interquartile range), and categorical variables were presented as number and proportion. Differences between groups were compared using an independent samples *t* or Wilcoxon’s rank sum test for continuous variables and a chi-squared or Fisher’s exact test for categorical variables. *p* < .05 was considered statistically significant. Diagnostic accuracy was expressed through sensitivity, specificity, and consistency.

## Results

### Participant characteristics

There were 355 males and 282 females with an average age of 46.49 ± 11.32 years and a median dialysis age of 84.0 (60.0, 120.0) months. All patients received regular hemodialysis three times a week or daily peritoneal dialysis (591 hemodialysis cases and 46 peritoneal dialysis cases). The operation success rate was 89.32% (569/637). Among the 569 patients with successful operations, 175 PTX patients were enrolled in group A, and 394 patients were enrolled in group B (Figure 1), which met the minimum sample size requirements. No significant difference was found in the general status of patients between the two groups before surgery (Table 1).

**Table 1.** Clinical characteristics and laboratory results.

Variable	Group A (*n* = 175)	Group B (*n* = 394)	*p*
Demographics			
Age (y)	46.99 ± 10.63	46.63 ± 11.40	.717
Gender (male/female)	92/83	225/169	.315
Height (cm)	164.08 ± 9.07	165.10 ± 8.60	.203
Weight (Kg)	58.53 ± 12.45	60.42 ± 11.51	.081
BMI (kg/m^2^)	21.62 ± 3.45	22.12 ± 3.59	.129
SBP (mmHg)	138.75 ± 19.90	139.03 ± 19.56	.877
DBP (mmHg)	84.66 ± 11.24	85.82 ± 12.38	.293
Type of dialysis, *n* (%)			
HD	167 (95.43%)	361 (91.62%)	.105
PD	8 (4.57%)	33 (8.38%)	.105
Duration of dialysis (m)	96.00 (60.00, 120.00)	84.00 (60.00, 120.00)	.378
Cause of ESRD, *n* (%)			
Chronic glomerulonephritis	155 (88.57%)	332 (84.26%)	.177
Diabetic nephropathy	2 (1.14%)	3 (0.76)	1.000
Hypertensive nephropathy	2 (1.14%)	14 (3.55%)	.183
Polycystic kidney	6 (3.43%)	17 (4.31%)	.620
Other	10 (5.71%)	28 (7.11%)	.539
Laboratory values			
Calcium (mmol/L)	2.57 ± 0.24	2.55 ± 0.44	.412
Phosphorus (mmol/L)	2.16 ± 0.57	2.19 ± 0.50	.285
AKP (u/L)	380.80 (169.20, 835.93)	318.00 (178.48, 739.03)	–
ln AKP	5.89 ± 0.94	5.85 ± 0.90	.604
Calcium (mmol/L)	2.57 ± 0.24	2.55 ± 0.44	.412
Phosphorus (mmol/L)	2.16 ± 0.57	2.19 ± 0.50	.285
Preoperative iPTH (pg/mL)	2008.30 (1363.10, 2721.50)	1925.95 (1320.10, 2684.68)	.238
D1-iPTH (pg/mL)	9.50 (5.10, 19.30)	8.95 (5.20, 15.63)	.232
D4-iPTH (pg/mL)	5.40 (2.30, 13.70)	5.70 (3.00, 17.65)	.500

Data were mean ± standard deviation (SD), or numbers and percentages, or median (25–75th percentile), as appropriate. Significance between the two groups were obtained from Independent-Samples *t* test or Wilcoxon’s rank sum test for continuous variables and Chi-square test or Fisher’s exact test for categorical variables.

BMI: body mass index; SBP: Systolic blood pressure; DBP: Diastolic blood pressure; HD: Hemodialysis; PD: Peritoneal dialysis; ESRD: end stage renal disease; AKP: alkaline phosphatase; iPTH: intact parathyroid hormone; D1-iPTH: iPTH level on the first day after surgery; D4-iPTH: iPTH level on the fourth day after surgery.

### Preoperative parathyroid imaging and postoperative parathyroid pathology in the two groups

The sensitivity, specificity, and diagnostic consistency of the two groups were compared in Table 2. The sensitivity and diagnostic consistency of group B were higher than those of group A (*p <* .001). There was no significant difference of specificity between the two groups.

**Table 2.** Preoperative imaging and postoperative parathyroid pathology results of two groups.

	Group A (*n* = 175)					Group B (*n* = 394)				
	RU	RL	LU	LL	Total	RU	RL	LU	LL	Total
TP	83	128	105	124	440	263	334	299	316	1212
FP	1	3	2	5	11	4	9	1	3	17
FN	83	38	65	38	224	116	37	84	59	296
TN	8	6	3	8	25	11	14	10	16	51
Se (%) (95%CI)	50.0 (42.2–57.8)	77.1 (70.0–83.3)	61.8 (54.0–69.1)	76.5 (69.3–82.8)	66.3 (62.5–69.9)	69.4 (64.5–74.0)	90.0 (86.5–92.9)	78.1 (73.6–82.1)	84.3 (80.2–87.8)	80.4 (78.3–82.3)
Sp (%) (95%CI)	88.9 (51.8–99.7)	66.7 (29.9–92.5)	60.0 (14.7–94.7)	61.5 (31.6–86.1)	69.4 (51.9–83.7)	73.3 (44.9–92.2)	60.9 (38.5–80.3)	90.9 (58.7–99.8)	84.2 (60.4–96.6)	75.0 (63.0–84.7)
PLR (95%CI)	4.50 (0.71–28.74)	2.31 (0.92–5.85)	1.54 (0.52–4.54)	1.99 (0.99–3.98)	2.17 (1.32–3.56)	2.60 (1.12–6.04)	2.30 (1.38–3.83)	8.59 (1.32–55.69)	5.34 (1.89–15.09)	3.22 (2.13–4.86)
NLR (95%CI)	0.56 (0.43–0.74)	0.34 (0.20–0.59)	0.64 (0.30–1.34)	0.38 (0.23–0.64)	0.49 (0.38–0.62)	0.42 (0.30–0.59)	0.16 (0.11–0.26)	0.24 (0.18–0.32)	0.19 (0.14–0.25)	0.26 (0.22–0.31)
CR (%) (95%CI)	52.00 44.3–59.6	76.57^a^ 69.6–82.6	61.7^a^ 54.1–68.9	75.43^b^ 68.4–81.6	66.43 62.8–69.9	69.54^c^ 64.7–74.1	88.32^ac^ 84.7–91.3	78.43^c^ 74.0–82.4	84.26^bc^ 80.3–87.7	80.14^d^ 78.1–82.1

FN: False Negative; FP: False Positive; TN: True Negative; TP: True Positive; Se: Sensitivity; Sp: Specificity; PLR: Positive Likelihood Ratio; NLR: Negative Likelihood Ratio; CR: Concordance rate; RU: right upper gland; RL: right lower gland; LU: left upper gland; LL: left lower gland.

^a^RL compared with RU in the same group, *p* < .001.

^b^LL compared with LU in the same group, *p* < .05.

^c^RU, RL, LU and LL in group B compared with of group A, *p* < .05.

^d^Total sensitivity and concordance rate in group B compared with group A, *p* < .001.

The sensitivity and diagnostic consistency for the upper parathyroid glands were lower than for the lower parathyroid glands in both groups (RU vs. RL and LU vs. LL, *p <* .05). There was no significant difference of specificity among all subgroups.

### Comparison of sensitivity for eutopic and ectopic parathyroids between the two groups

The sensitivity of preoperative eutopic and ectopic parathyroid imaging was compared between the two groups (Table 3). Nine patients (9/175, 5.14%) had ectopic parathyroid glands in group A, and 20 patients (20/394, 5.08%) had ectopic parathyroid glands in group B. As shown in Table 3, there was no difference in the incidence and sensitivity of preoperative ectopic parathyroid imaging between the two groups, while a significant difference in sensitivity was found between the two groups in preoperative eutopic parathyroid imaging (*χ*^2^ = 50.378, *p* < .001).

**Table 3.** Comparison of eutopic and ectopic parathyroids between the two groups.

	Group A (*n* = 175)	Group B (*n* = 394)
Eutopic parathyroids		
TP	440	1212
FP	11	17
FN	224	296
Se (%) (95%CI)	66.3 (62.5–69.9)	80.4^a^ (78.3–82.3)
Ectopic parathyroids		
TP	9	19
FP	0	1
FN	2	2
Se (%) (95%CI)	81.8 (48.2–97.7)	90.5 (69.6–98.8)

TP: True Positive; FP: False Positive; FN: False Negative; Se: Sensitivity.

^a^Group B compared with group A, *p* < .001.

### Planar and SPECT/CT fusion imaging of preoperative parathyroids findings

**Figure 2.**
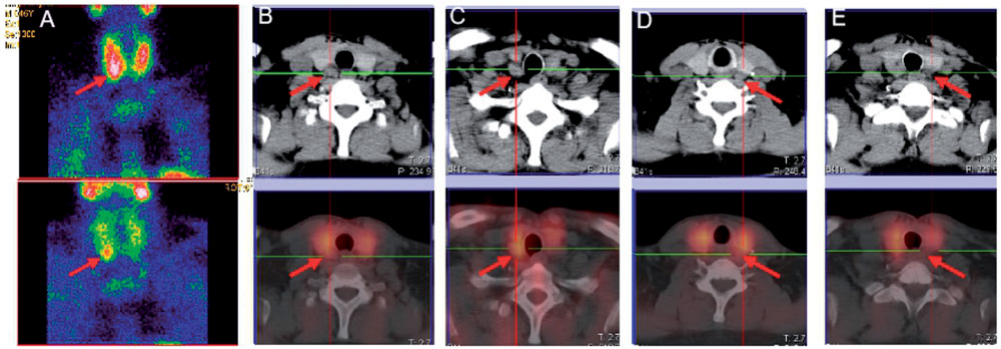
Comparison of planar imaging and SPECT/CT fusion imaging of a 46-year-old man in group B.

A comparison of planar imaging and SPECT/CT fusion imaging is shown in Figure 2, which displays images of the same patient in group B. Figure 2(A) is a dual-phase ^99m^Tc-MIBI plane image that shows the lower pole of the right lobe of the thyroid gland, suggesting that there was only one hyperplastic parathyroid gland. In Figure 2(B–E), SPECT/CT was combined in the early phase of ^99m^Tc-MIBI, and the resulting images show four parathyroid glands lying on the dorsal side of the thyroid gland. In contrast to Figure 2(B–E), Figure 2(A) missed three hyperplastic parathyroid glands, suggesting that the SPECT/CT tomographic images increased the diagnostic positive rate compared to ^99m^Tc-MIBI alone.

**Figure 3.**
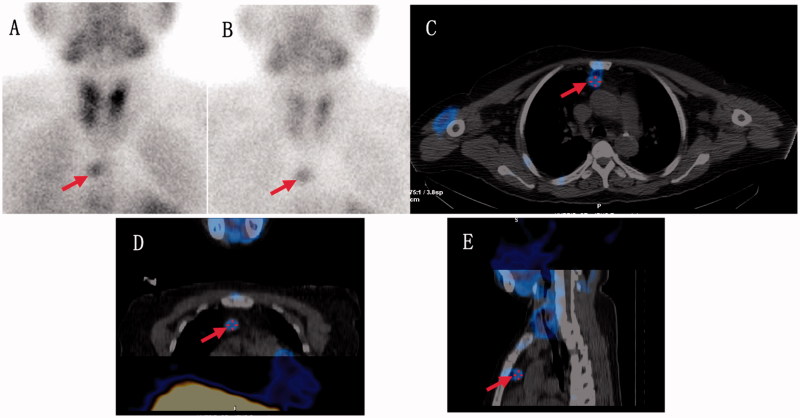
A 55-year-old woman with anterior mediastinum ectopic parathyroid.

Figure 3 shows images of the same patient in group B. Figure 3(A,B) (early phase and delayed phase planar imaging, respectively) showed anterior mediastinum ectopic parathyroid. Figure 3(C–E) (transverse, coronal, and sagittal SPECT/CT fusion imaging, respectively) showed the same results as Figure 3(A,B), but the locations of parathyroid glands were more accurate.

## Discussion

SHPT is a common complication in end-stage renal disease, which affects mostly patients on maintenance dialysis. It is followed by disorders of calcium and phosphorus metabolism, parathyroid hyperplasia, and abnormal PTH secretion [18]. Numerous studies have confirmed that SHPT leads to mineral metabolic disorders, fractures, and cardiovascular disease, resulting in increased mortality in patients with chronic kidney disease. The National Kidney Foundation Kidney Disease Outcomes Quality Initiative (KDOQI) suggested parathyroidectomy to treat severe secondary hyperparathyroidism when drug regimes fail [16].

Preoperative localization of the hyperplastic parathyroid glands is the most important factor for the success of the operation. Ultrasonography, one of the earliest used techniques, is noninvasive, inexpensive, and still widely used today. Parathyroid nodules should be distinguished from thyroid nodules, since failure to do so is the most common cause of false positives. Parathyroid glands are small in size, varying in position (from the carotid sheath down to the mediastinum), and the rate of glands located in the blind area of B-ultrasound in the neck is around 5–10% [19]. Sonographers often find it more challenging to detect the glands in recurrent SHPT patients as the neck anatomy differs from the previous surgery. ^99m^Tc-MIBI dual-phase imaging is a functional imaging technique that is not disrupted by the above factors, and hyperfunctional parathyroid tissue can be found in most cases. Furthermore, SPECT/CT fusion images can provide accurate locations of lesions along with clear three-dimensional images and their relationship with adjacent tissues, thus helping surgical procedures [20]. Therefore, dual-phase ^99m^Tc-MIBI imaging technology has been used more widely during recent years. Unlike ultrasound and CT, ^99m^Tc-MIBI imaging is not only an imaging examination but also a functional imaging tool, which is less affected by the anatomic variation and now the most commonly used in practice. Recently, a meta-analysis of 471 patients including 24 studies indicated that the sensitivity of MIBI in detecting SHPT proliferative glands was 58% [21]. Vulpio et al. reported that MIBI combined with ultrasound amplified the sensitivity of accuracy up to 73% [22].

In this study, by analyzing the results of dual-phase ^99m^Tc-MIBI imaging with pathology, we found that the sensitivity of the upper two parathyroid glands was significantly lower than the lower glands in both groups, which is consistent with our previous research and other studies [22,23]. This phenomenon occurs because of false-negatives due to high variations in the anatomical positions of the superior parathyroid glands [23] or due to the superior parathyroid glands being hidden by the thyroid gland. In addition, our results showed that sensitivity and diagnostic consistency increased significantly in group B compared with group A, suggesting that the sensitivity and diagnostic consistency of the upper two parathyroid glands increased. Although there may be some factors to improve the diagnosis, such as the possibility of experienced radiologists refining the reading level, it still suggests that CT scans can increase the sensitivity and consistency of all parathyroid glands, especially for the upper two parathyroid glands, which will help in surgical exploration by reducing the time in finding the parathyroid tissue and thus shortening the operation time [21] and decreasing the possibility of recurrent laryngeal nerve or other adjacent structure damage.

Our observation also found that the success rate of surgery was 89.32%. The possible reasons of operation failure were some small parathyroid glands that were difficult to find in operations, false-negatives of detection, and the special locations of ectopic parathyroid glands, which were also difficult to find. Besides, there was no statistical difference of the specificity between groups A and B in our study, probably due to the small number of true negative cases. This is because most of the parathyroid glands were hyperplastic in the patients with severe SHPT that met the surgical indications in this study.

A major strength of this study is that it was conducted in consecutive cases to reduce selection bias. From March 2010 to June 2013 patients all received dual-phase ^99m^Tc-MIBI planar imaging, and from July 2013 to December 2016 patients received dual-phase ^99m^Tc-MIBI planar imaging plus early phase SPECT/CT. To reduce misclassification errors in case ascertainment, interpretation of planar images or SPECT/CT images was performed in consensus by two experienced nuclear medicine physicians. One potential limitation of this study is that we still have to take into consideration the abilities of the radiologists, the surgeon’s increased operative proficiency and other factors.

In short, ^99m^Tc-MIBI SPECT/CT fusion imaging is the latest radionuclide imaging technology, and it provides the perfect combination of functional and anatomical information. This study shows that ^99m^Tc-MIBI SPECT/CT technology can effectively fuse anatomical images, increasing the detection rate of eutopic parathyroid glands, especially for upper parathyroid glands. For ectopic parathyroids, the detection rate cannot be increased, but accurate anatomical locations of the glands can be found, which is useful for efficiency improvement and surgery safety.
